# The values of HER-2 expression in the non-muscle-invasive bladder cancer: a retrospective clinical study

**DOI:** 10.3389/fonc.2023.1243118

**Published:** 2023-11-27

**Authors:** Shuo Wang, Yongpeng Ji, Yiqiang Liu, Peng Du, Jinchao Ma, Xiao Yang, Ziyi Yu, Yong Yang

**Affiliations:** ^1^ Key Laboratory of Carcinogenesis and Translational Research (Ministry of Education), Urological Department, Peking University Cancer Hospital & Institute, Beijing, China; ^2^ Key Laboratory of Carcinogenesis and Translational Research (Ministry of Education), Department of Pathology, Peking University Cancer Hospital & Institute, Beijing, China

**Keywords:** non-muscle-invasive bladder cancer, human epidermal growth factor receptor 2 (HER-2), antibody-drug conjugate, ROC curve analyses, IHC

## Abstract

**Purpose:**

The purpose of this research is to evaluate the association between HER-2 expression and clinicopathological features in patients with non-muscle-invasive bladder cancer (NMIBC).

**Methods:**

Between 2019 and 2022, 204 patients treated with Transurethral resection of the bladder tumor (TURBT) were included in this study. Data of pathologic T (pT) stage, grades of the tumor, age, sex, tumor size and number of the tumors were collected and compared according to the expression level of the human epidermal growth factor 2 (HER-2). ROC curve analysis was performed to assess the discriminative ability of HER-2 expression for tumors grades and pT stage. Multivariable logistic regression analysis were used to evaluate the association between HER-2 expression and tumor grades and pT stage.

**Results:**

Patients were divided into low grade (110, 53.9%) and high grade groups (94, 46.1%) according to the tumor grade. Pathologic stage consisted of pTa in 166 (81.4%) and pT1 in 38 (18.6%). HER-2 expression was semi quantitatively scored to 0 in 44 (21.6%), 1 in 58 (28.4%), 2 in 91 (44.6%), and 3 in 11 (5.4%) cases. HER-2 expression was significantly associated with tumor stages and histological grades, but not with sex, tumor size or number of tumors. The AUC for combination of HER-2 expression with tumor stages and histological grades was 0.652 (p < 0.003) and 0.727 (p < 0.001), respectively.

**Conclusion:**

This study demonstrated that HER-2 expression is associated with tumor stages and histological grades in NMIBC. It has diagnostic value for cystoscopic biopsy.

## Introduction

Urinary carcinoma of bladder (UCB) is the ninth of the most common cancer in the world (1), Muscle-invasive bladder cancer (MIBC) and non-muscle-invasive bladder cancer (NMIBC) have completely different treatment strategies. NMIBC account for 70–75%, while the remaining 25–30% are MIBC (2). The tumor recurrence of NMIBC varies from 30% to 80%, furthermore, in 10% to 20% of patients the disease will progress to MIBC (3). However, high risk NMIBC in particular remains a challenging tumor to treat. High recurrence rates in NMIBC means patients are exposed to frequent hospital visits. Transurethral resection of the bladder tumor (TURBT) is the main procedure for treatment and diagnosis of UCB. Bacillus Calmette Guerin (BCG) still remains the gold standard for adjuvant treatment of high risk NMIBC patients (3, 4). BCG therapy is challenging in terms of its ineffectiveness and adverse effects. 35% patients who were tumor free at 2 years had recurrent tumors during the 2 to 11 years follow up (5). Surgical removal of the bladder should be considered in case of BCG unresponsive tumors or in NMIBC with the highest risk of progression. 15% of patients who discontinue treatment after first course of BCG treatment, 35% of them are known to have difficulty in continuing treatment due to side effects of bacterial or chemical cystitis, hematuria, and systemic febrile events (6).

The human epidermal growth factor receptor 2 (HER-2) expression has been extensively investigated as a target therapy and is known to be a prognostic and therapeutic marker in breast cancer. overexpression of HER-2 in 17% to 76% of cases has been reported in UCB, and there is a possible association between HER-2 overexpression with early recurrence, high grade disease, and worse prognosis (7–9).Before the development of antibody-drug conjugate (ADC), human epidermal growth factor receptor-2 (HER-2) received attention as a therapeutic target but was shown to be ineffective. Therefore, the predictive value of HER-2 status in bladder cancer remains controversial (10). Moreover, there are only a few studies of HER-2 status in NMIBC (11–13). Promising results in targeted therapy comes from novel ADC agents, ADCs bind to tumor associated antigens, triggering endocytosis, internalization, and release of the cytotoxic payload in target tumor cells after lysosomal degradation. There is an opportunity to develop an intravesical therapeutic agent for this identified target antigen by mediating ADCs, for example, RC48 using to develop a HER-2-targeting ADC for the treatment of mUCB (14). Meanwhile, here have been several advancements in UCB treatment (15, 16). To determine the prognostic values of HER-2 expression in bladder cancer, we recently analyzed the association between HER-2 expression and clinicopathological features in patients with non-muscle-invasive bladder cancer (NMIBC).

## Materials and methods

### Patients’ characteristics

This retrospective study was approved by the Institutional Review Board of Peking University Cancer Hospital and Institute according to the principles of the Declaration of Helsinki. The requirement for informed consent was waived due to the retrospective nature of the study. In this study, we retrospectively analyzed clinical pathological data from 204 patients treated with TURBT at the Department of Urology of the Peking University Cancer Hospital between 2019 and 2022 who were initially diagnosed with NMIBC. They were diagnosed as pTa or pT1 papillary urothelial tumors based on primary TURBT, according to the 7th edition American Joint Committee on Cancer TNM system. The exclusion criteria were diagnosis of muscle invasive carcinoma of the bladder (≥pT2),the presence of carcinoma *in situ* (CIS),remaining tumor after initial resection, metastatic disease, concomitant diagnosis of another cancer. All patients did not receive treatment before TURBT and had no history of chemotherapy or radiotherapy. All patients had an immediate (within 8 hours) post-operation single intravesical treatment with pirarubicin (THP).

### Immunohistochemistry

**Figure 1:**
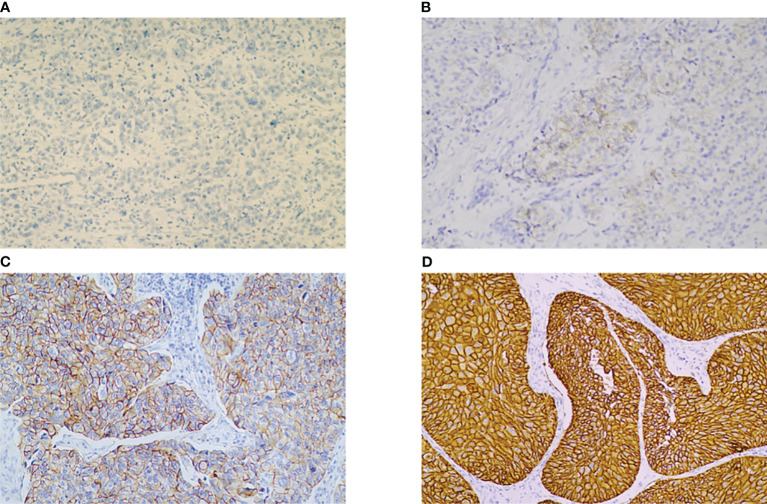
IHC for HER-2 showing 0, no staining **(A)**, 1+, faint/barely partial membrane staining less than 50% of tumor area. **(B)**, 2+, variable weak-to-moderate complete membrane staining in ≥ 50% of tumor area **(C)**, 3+ moderate to strong complete membrane staining in all tumor area. **(D)** (magnification × 200). HER-2: human epidermal growth factor receptor-2.

Formalin-fixed, paraffin-embedded tumor samples from specimens of 204 TURBT patients were collected. All specimens were examined in the pathology department of Peking University Cancer Hospital. Confirmation of T stage, tumor grade, and HER-2 expression were assessed by a single pathologist, and conducted a re-examined. The scoring was first semi quantitatively analyzed and grouped into 4 categories as follows: 0, no staining; 1+, faint/barely partial membrane staining less than 50% of tumor area; 2+, variable weak to moderate complete membrane staining in ≥ 50% of tumor area; 3+, strong complete membrane staining in all tumor area (**Figure 1**).

### Statistical analysis

Descriptive statistics were used to summarize patient characteristics, categorical variables were presented as numbers and percentages, and continuous variables were presented as the mean and standard deviation. The chi-square or Fisher exact test was used to evaluate the statistical significance of the associations between clinicopathologic parameters. ROC curve analyses were performed to assess the discriminative ability of the HER-2 combined with tumor grade. The cutoff points for markers were defined by a criterion based on Youden’s index defined as YI(C) = max c [Se(C) + SP(C)-1] and corresponding specificity–sensitivity levels were provided. The binary logistic regression model (multivariate analysis) was used to evaluate the association between clinicopathological characteristics and HER-2 expression. A p value of < 0.05 (two-tailed) was used to establish statistical significance. All statistical analyses were conducted using SPSS v. 19.0 (SPSS Inc., Chicago, IL, USA).

## Results

The clinicopathological characteristics of the 204 patients with NMIBC are summarized in **Table 1**. The patients included 161 men (78.9%) and 43 women (21.1%). The mean age of the patients was 61.6 yr (range 30-88 yr). Among them, the mean age of male patients was 61.5 yr (range 30-88 yr),and the mean age of female patients was 62.1 yr (range 34-88 yr). The number of patients with tumors smaller than 3cm is 190(93.1%).3 cm or more sized tumor was found in 14 (6.9%). Patients with one tumor bladder was found in 138 cases (67.6%), and 2 or more of multifocality was found in 66 cases (32.4%). Patients were divided into low grade (110, 53.9%) and high grade groups (94, 46.1%) according to the tumor grade. Pathologic stage consisted of pTa in 166 (81.4%) and pT1 in 38 (18.6%).HER-2 expression was semi quantitatively scored to 0 in 44 (21.6%), 1 in 58 (28.4%), 2 in 91 (44.6%), and 3 in 11 (5.4%) cases.

**Table 1:** Clinicopathological characteristics of 204 patients with NMIBC.

Characteristics	No. (%) of cases
Sex	
Male	161 (78.9)
Female	43 (21.1)
Age (mean, range, yrs)	61.6 (30-88) ± 11.1
Male	61.5 (30-88) ± 11.0
Female	62.1 (34-88) ± 11.6
pT stage	
Ta	166 (81.4)
T1	38 (18.6)
Tumor grade	
Low grade	110 (53.9)
High grade	94 (46.1)
HER2 protein expression (IHC)	
0	44 (21.6)
1+	58 (28.4)
2+	91 (44.6)
3+	11 (5.4)
Number of tumors	
Single	138 (67.6)
2-7	54 (26.5)
≥8	12 (5.9)
Tumor size	
< 3 cm	190 (93.1)
≥ 3 cm	14 (6.9)

NMIBC, non-muscle invasive bladder cancer; HER-2, human epidermal growth factor receptor-2.

Pathologic stage was significantly associated with HER-2 (P = 0.020), tumor grade (P < 0.001), Gross tumor appearance was evaluated. The proportion of patients with low grade was higher in the tumors< 3 cm group than in the tumors≥ 3 cm group (P = 0.011), but not with HER-2 (P = 0.402) or pathologic stage (P = 0.089). The proportion of patients with low grade was higher in single tumor group than in the 2-7 tumors group and higher in the ≥8 tumors group than in the 2-7 tumors group (P = 0.006).And the proportion of patients with pTa tumors was higher in single tumor group than in the 2-7 tumors group and higher in the ≥8 tumors group than in the 2-7 tumors group (P = 0.021) (**Table 2**, **3**).

**Table 2:** Comparison of clinicopathologic parameters according to the HER-2 expression.

Parameters Total (n = 204)	HER2 IHC				P value
	0	1+	2+	3+	
Tumor grade					
ow	36	38	36	0	< 0.001
High	8	20	55	11	
pT stage					
Ta	41	50	68	7	0.020
T1	3	8	23	4	
Sex					
Male	38	44	71	8	0.554
Female	6	14	20	3	
Tumor size					
< 3 cm	42	55	84	9	0.402
≥ 3 cm	2	3	7	2	
Number of tumors					
Single	36	40	58	4	0.119
2-7	7	15	26	6	
≥8	1	3	7	1	

NMIBC, non-muscle invasive bladder cancer. HER-2, human epidermal growth factor receptor-2.

**Table 3:** Comparison of clinicopathologic parameters according to the tumor grades or pT stage.

Parameters Total (n = 204)	Tumor grade		P value	pT stage		P value
	Low grade	High grade		Ta	T1	
Tumor grade						
Low	NA	NA	NA	106	4	< 0.001
High	NA	NA	NA	60	34	
Sex						
Male	81	80	0.045	130	31	0.656
Female	29	14		36	7	
Tumor size						
< 3 cm	107	83	0.011	157	33	0.089
≥ 3 cm	3	11		9	5	
Number of tumors						
Single	85	53	0.006	116	22	0.021
2-7	20	34		38	16	
≥8	5	7		12	0	

NMIBC, non-muscle invasive bladder cancer; pT stage, pathologic T stage; NA, Not Applicable.

**Figure 2:**
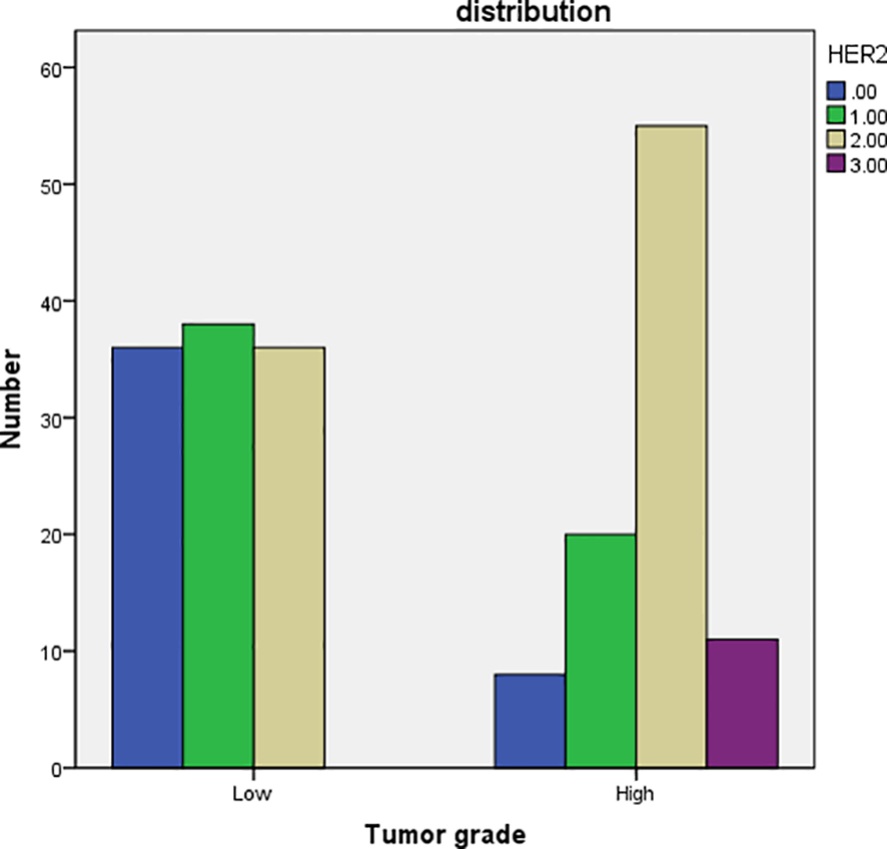
the bar chart of comparison of tumor grade with HER-2 IHC. HER-2: human epidermal growth factor receptor-2.

**Figure 3:**
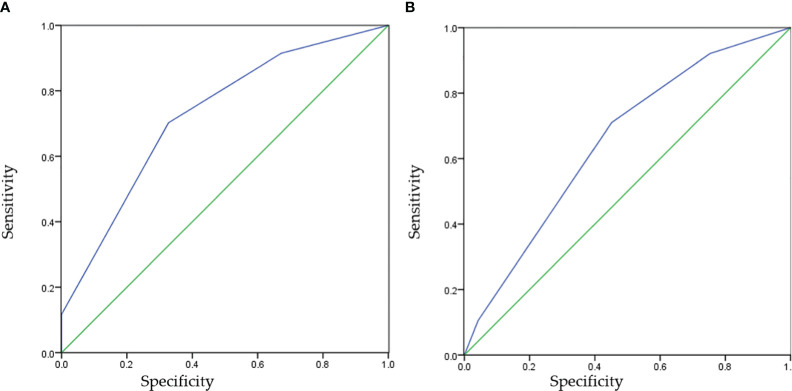
The receiver operating characteristic curves of HER-2 IHC for predicting tumor grades or pT stage. HER-2, human epidermal growth factor receptor-2. **(A)** Specificity=False Positive Rate, **(B)** Sensitivity=True Positive Rate.

In the low grade group the HER-2 expression has a similar proportion in 0,1+ and 2+, but there was no 3+. However, there was a high proportion of 2+and3+ HER-2 expression in high grade group (**Figure 2**). We classified patients and assessed clinical usefulness of HER-2 expression + tumor grade, and HER-2 expression + pathologic stage. The results revealed that HER2-positive could distinguish patients with high grade from low grade (AUC = 0.743,p < 0.001) with a sensitivity of 91.5% and a specificity of 67.3%, with a threshold value of 0.5; and HER2-positive could distinguish patients with pT1 tumors from pTa tumors (AUC = 0.652, p = 0.003) with a sensitivity of 92.1% and a specificity of 75.3%, with a threshold value of 0.5 (**Figure 3**, **Table 4**). There was a prognostic difference between HER 2 non expression and HER2 expression. We chose HER-2 0 and HER expression as different groups. Multivariable logistic regression models were used to evaluate the association between HER-2 and clinical characteristics. We found that HER-2 was only related to tumor grade (**Table 5**).

**Table 4:** Cut-off, AUC, sensitivity and specificity values of HER-2 for pT stage and tumor grades.

Variables	AUC	Cut-off	Sensitivity	Specificity	95% CI	P values
tumor grade	0.727	0.5	91.5%	67.3%	0.658-0.796	< 0.001
pT stage	0.652	0.5	92.1%	75.3%	0.560-0.744	0.003

HER-2, human epidermal growth factor 2; pT stage, pathologic T stage.

**Table 5:** multivariable analyses of HER-2 expression for predicting tumor grades, pT stage.

	HER-2 (-) vs HER-2 (+)		
	OR	95% CI	P value
Tumor grade			
Low	1.00	1.848-11.774	0.001
High	4.665		
Pathologic stage			
Ta	1	0.419-6.500	0.473
T1	1.651		
Sex			
Female	1	0.134-0.960	0.141
Male	0.358		
Tumor size			
< 3 cm	1	0.175-4.935	0.931
≥ 3 cm	0.929		
Number of tumors			
Single	1		
2-7	0.275	0.032-2.346	0.238
≥8	0.513	0.053-4.952	

HER-2(-) was defined as HER-2 0 expression; HER-2 (+) was defined as HER-2 1+, 2+ or 3+.

## Discussion

HER-2 is a member of the epidermal growth factor receptor family having tyrosine kinase activity. The role of HER-2 is known in breast cancer and gastric cancer because it is a prognostic factor and a classic therapeutic target when over expressed (17). Bladder cancer is characterized by the presence of two different subtypes: NMIBC and MIBC. MIBC has a poor outcome with common progression to metastasis, while NMIBC shows frequent recurrences.

The prognostic values of HER-2 expression in bladder cancer have remained unclear due to inconsistent results. Most of the studies of HER-2 expression in bladder cancer have been performed on MIBC and show varying expression ranging between 9% and 81% (18). Potential reason for this variation is the fact that studies used bladder cancer samples with a varying degree of tumor stages and histological grades. Nevertheless, there have been presented HER-2 over expression was significantly associated with poor clinicopathological factors including lymph node metastasis and poor prognosis in bladder cancer (8, 19, 20). MIBC with a luminal molecular subtype have been shown to have a significantly higher rate of HER-2 alterations than those of the basal subtype. There were higher HER-2 2/3+ expression (46%) in MIBC and high grade NMIBC (21).

However, reports of HER-2 status in NMIBC are limited. A few studies have shown HER-2 protein over expression and gene amplification in 4–12% and 3–8% of NMIBC (11–13), respectively. Although predicting such behavior is clinically important as recurrence and invasion bear a significant risk of metastasis and impaired survival. The European Association of Urology (EAU) implemented EORTC risk tables in its guidelines for predicting tumor recurrence and progression (3).

Several challenges in pathologic stage have hindered its adoption in clinical guidelines for NMIBC. Chen et al. (22) showed that HER-2 amplification could distinguish a subset of NMIBC patients with a high risk of disease progression. But Olsson et al. (13) reported HER-2 expression could not predict patient prognosis in a larger study that included 285 patients. Because the detection of concurrent CIS increases the risk of recurrence and progression of Ta and T1 tumors (3). We only select patients with Ta and T1 and exclude patients with CIS. In the present study, we found the expression of HER-2 was significantly different in pathological groups, low and high grade NMIBC, and pathologic stage, but not in tumor size or number of tumors. As we know, the pathologic stage assessed by the severity of invasion depth is one of the most important prognostic predictors of NMIBC (23).

These findings could explain that HER-2 over expression has been found to correlate with the incidence of recurrence and progression in NMIBC. In our study, HER-2 expression was demonstrated to be a reliable independent factor associated with non-muscle-invasive tumor grade. It can be used to predict the prognosis of tumor in cystoscopic biopsy.

The guidelines for NMIBC remain controversial; and conservative management may allow tumor to progress to muscle invasion, which requires cystectomy. There have been attempts to translate over expression of HER-2 in UCB into therapeutic modalities. As widely known, the therapy is currently used worldwide for treating breast and gastric cancers (24, 25). However, the results have not been as promising as in breast cancer (10). The recent emergence of novel ADCs has renewed the interest in HER-2 targeted therapies for UCB. They effectively kill tumor cells with minimal systemic side effects. ADCs bind to tumor associated antigens, triggering endocytosis, internalization, and release of the cytotoxic payload in target tumor cells after lysosomal degradation (26).RC48-ADC is a novel humanized anti-HER-2 antibody, impairing the formation of the microtubule network of target cells. However, reports on RC48-ADC in HER-2 2+ or HER-2 3+ UCB patients suggest that only a moderate level of protein expression is sufficient to induce a response. In patients with advanced urothelial carcinoma, Sheng et al. (14) reported the RC48-ADC is a promising efficacy with a manageable safety profile. One of the main challenges in treating solid tumors with ADCs is the heterogeneous expression of the target antigen (Ag) in primary tumor tissues. Cleavable linkers and hydrophobic payloads have been proposed as a possible solution, mainly because of their hypothetical capital role played in the so-called “bystander killing effect”, recently demonstrating promising clinical efficacy and preclinical activity even in HER-2 low tumors (27).

Although BCG is considered a very effective treatment for high risk NMIBC, consensus exists that not every patient with superficial bladder cancer should be treated with BCG due to its increased risk of toxicity (28). Meanwhile, urologists often have to treat patients not based on guidelines because of the chronic shortage of BCG and BCG failure remaining largely unknown (29). ADCs are promising intravesical drugs that directly exert cytotoxic effects on tumor cells by targeting specific tumor antigens, with reduced systemic adverse effects. As previously mentioned, the expression of HER2 was significantly different in pathological groups and pathologic stage.

## Conclusion

In summary, this study demonstrated that HER2 expression are associated with tumor stages and histological grades in NMIBC. It has diagnostic value for cystoscopic biopsy. and therapeutic strategies of HER2 expression could be considered for the management of NMIBC that tolerates BCG.

## Data availability statement

The original contributions presented in the study are included in the article/supplementary material. Further inquiries can be directed to the corresponding authors.

## Ethics statement

The studies involving humans were approved by Institutional Review Board of Peking University Cancer Hospital. The studies were conducted in accordance with the local legislation and institutional requirements. The human samples used in this study were acquired from primarily isolated as part of your previous study for which ethical approval was obtained. Written informed consent for participation was not required from the participants or the participants’ legal guardians/next of kin in accordance with the national legislation and institutional requirements.

## Author contributions

SW and PD designed the study. SW, YJ and YL made the same contribution in this study as the first coauthor. SW and PD made the same contribution in this study as the cor-corresponding authors. SW, YJ, YL, JM, XY, ZY, PD and YY performed the study and analyzed the data. PD, SW, YJ and YL wrote the manuscript draft and revised the manuscript. All authors have read and agreed to the published version of the manuscript.
